# Os Acromiale: Reviews and Current Perspectives

**DOI:** 10.1111/os.12518

**Published:** 2019-09-05

**Authors:** Tian You, Simon Frostick, Wen‐tao Zhang, Qi Yin

**Affiliations:** ^1^ Sports Medicine Department Peking University Shenzhen Hospital Shenzhen China; ^2^ Department of Orthopaedic Surgery Royal Liverpool University Hospital Liverpool UK

**Keywords:** Arthroscopy, Os acromiale, Reverse shoulder arthroplasty, Rototar cuff tear, Shoulder impingement

## Abstract

Os acromiale is a developmental defect which results from the lack of an osseous union between the ossification centers of the acromion, leading to the fibrocartilaginous tissue connection. The prevalence of os acromiale is 1% to 15%, and is quite common in the African American population. Os acromiale in adults is easily diagnosed by symptoms and X‐ray, particularly on the axillary view; however, the differential diagnosis of adolescents may require MRI or SPECT–CT. Generally, nonoperative therapy for symptomatic os acromiale should be started, including physiotherapy, nonsteroidal anti‐inflammatory drugs, and injections. Surgical treatment is indicated after failed conservative treatment. In symptomatic patients with fixable acromiale, the tension band technique should be used to make the anterior aspect of the acromion elevated from the humerus head. In patients with small fragments which are unsuitable for reattachment, excision might be the best therapeutic option and lead to good outcomes. Whether using internal fixation or resection, the arthroscopic technique results in a better outcome and fewer complications, especially in older patients or athletes with overhead movement, because of the high incidence of shoulder impingement or rotator cuff tears which can be treated concurrently.

## Introduction

Os acromiale represents an unfused accessory center of ossification of the acromion of the scapula. It is regarded as one of the reasons for rotator cuff tears and shoulder impingement^1^, ^2^, which is generally asymptomatic and discovered accidentally^3^, ^4^. This anatomic deformity occurs more frequently in persons of Black ancestry than in persons of White, Native American and Middle Eastern ancestries^5^. Treatment for symptomatic patients is primarily non‐operative, like nonsteroidal anti‐inflammatory drug, physical therapy or corticosteroid injection. Surgical procedures are typically recommended only after non‐operative treatments have failed. Common procedures include arthroscopic subacromial decompression with acromioplasty^6^, open or arthroscopically assisted reduction and internal fixation with or without bone grafting^7^, ^8^, and open or arthroscopic excision of the os fragment^9^.

## Method of Searching

The inclusion criteria was studies related to os acromiale. Exclusion criteria were: (i) papers not in English; and (ii) commentary or letters to the editor.

The purpose of this study is to review reports on topics related to os acromiale that have been published in PubMed (from 1984 to May 2018). We used the key word “os acromiale” to query the PubMed database of the US National Library of Medicine. From the resulting list, we reviewed 101 published papers. We categorized, summarized, and included 52 studies finally.

### *Anatomy*

**Figure 1:**
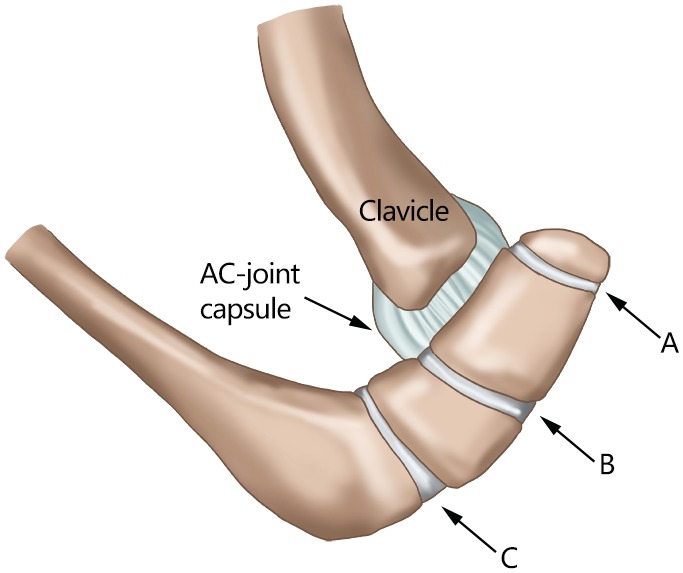
The types of os acromiale: (A) the space between the os pre‐acromiale and the acromion; (B) the space between the os meso‐acromiale and the acromion (note there is a communication with the acromioclavicular joint, found in all our patients); and (C) the space between the os meta‐acromiale and the acromion^14^.

The acromion is normally formed by the fusion of several ossification centers^10^. Macalister^11^ found that several ossification points fuse to form three major elements. The anterior element is the preacromion, the middle element is the mesacromion, and the posterior element, which forms the acromial angle, is the metacromion. These three elements merge to form a triangular epiphyseal bone, which finally fuses with the basiacromion. The basiacromion typically fuses with the scapular spine by age 12, and all four centers should unite by ages 15 to 18. However, some do not have complete ossification until as late as age 25 years^12^, leading to an inhomogeneous group of variations known as “os acromiale.” Thus, any diagnosis of such deformity should not be defined until after this time point^13^. The types of os acromiale are determined according to the unfused segment immediately anterior to the site of nonunion^14^, ^15^, which contains the meta‐acromion (base), the meso‐acromion (mid), and the pre‐acromion (tip) from proximally to distally (Fig. 1)^14^.

### *Prevalence*

#### *Morbidity*

The frequency of os acromiale has ranged from 1 to 15% in radiographic and anatomical studies^9^, ^11^, ^16^, ^17^, ^18^. Case *et al*.^19^ compared a South African cadaver sample (*n* = 494) with a medieval Danish archaeological sample (n = 532). The results showed that the South African frequency (18.2%) was significantly higher than the medieval Danish frequency (7.7%, *P* < 0.0001, and a left side bias (72%) among the South Africans (*P* = 0.013). However, sex and age biases were not found. Kumar *et al*.^20^ reviewed the X‐rays and MRI of Korean patients visiting a shoulder clinic, and found that 13 cases out of 1568 patients had an os acromiale; there were 5 and 8 cases of pre‐acromiale and meso‐acromiale, respectively. Thus, the prevalence of os acromiale in this study population was found to be 0.7 (7 cases per 1000 patients), which is much lower than for Black and White people, comparing with other studies. At the same time, gender and hand dominance was not associated with frequency of os acromiale. A systematic review and meta‐analysis of 23 studies revealed a significantly higher frequency in persons of Black ancestry than in persons of White, Native American, and Middle Eastern ancestries, and significantly higher unilateral and bilateral frequencies in those with Black ancestry; there were no significant interactions of Os acromiale frequency with gender and side^5^.

#### *Os Acromiale with Rotator Cuff Tear*

**Figure 2:**
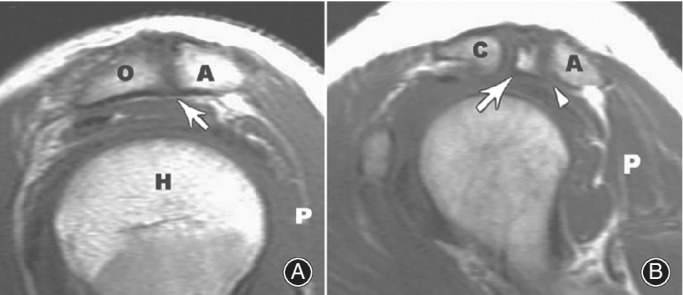
(A) Os acromiale without step‐off deformity. T1‐weighted sagittal MRI of the shoulder shows an os acromiale without step‐off deformity (arrow) relative to the inferior cortex of the acromion. A, acromion; H, humeral head; O, os acromiale; P, posterior. (B) Os acromiale with step‐off deformity. T1‐weighted sagittal MRI of the shoulder shows malalignment (white arrowhead) between the inferior cortex of the os acromiale (arrow) and the acromion. A, acromion; C, clavicle; P, posterior^24^.

Some previous studies have demonstrated a high incidence of full thickness rotator cuff tears in os acromiale patients (approximately 50%)^21^, ^22^. However, due to the small samples of the former papers, 15 and 6 shoulders, respectively, the latter studies re‐examine the relationship between rotator cuff tear and os acromiale. Boehm *et al*.^23^ assessed operation notes and axillary radiographs for the presence and the type of os acromiale in 1000 consecutive patients with open rotator cuff repairs. Only 62 patients (6.2%) revealed an os acromiale in the axillary radiographs, and the average number of tendons involved in the cuff tear was the same (1.4) in patients with and without os acromiale; the average age of patients with and without os acromiale was 55 and 56 years, respectively. This result was similar to the 8% general incidence of os acromiale reported by Yammine^5^. Ouellette *et al*.^24^ retrospectively analyzed 84 MRI studies of the shoulder, which revealed that the presence of os acromiale may not significantly predispose to supraspinatus and infraspinatus tendon tears. However, subjects with step‐off deformity (Fig. 2)^24^ of an os acromiale are at greater risk of rotator cuff tears than are similar subjects without such deformity. In young throwing athletes, Roedl *et al*.^25^ found that rotator cuff tears were significantly more common on the follow‐up MRI in patients with acromial apophysiolysis (68%, 15 of 22) compared with control patients (29%, 6 of 21; *P* = 0.015, Fisher exact test; OR = 5.4). In addition, grades of rotator cuff tears were significantly higher in patients with acromial apophysiolysis compared with control patients (*P* = 0.03).

In general, the most common os acromiale is the large, relatively triangular mesoacromion, which forms an interface with the acromion in proximity to the acromioclavicular joint (ACJ), while a less common os acromiale, the preacromion, is noted at the distal tip of the acromion^26^. There is a strong relationship between os acromiale and race, while the correlation between os acromiale and rotator cuff tear is full of controversies.

### *Symptoms and Diagnosis*

Os acromiale can be easily diagnosed with plain X‐rays with at least two views (AP, axillary views). As mentioned above, the frequency of os acromiale has ranged from 1 to 18.2% in radiographic and anatomical studies. If these figures are correct, clearly, most of patients with os acromial are asymptomatic.

#### *Symptoms*

Patients with symptomatic os acromiale, especially in young people and athletes with overhead activity, may have pain at the superior aspect of the shoulder^25^, ^27^. Symptoms can also occur at night. Along with pain, patients may have decreased shoulder motion and strength. Patients will be tender to palpation at the site of the os acromiale and there may also be obvious movement of the bone.

#### *Imaging Diagnosis*

**Figure 3:**
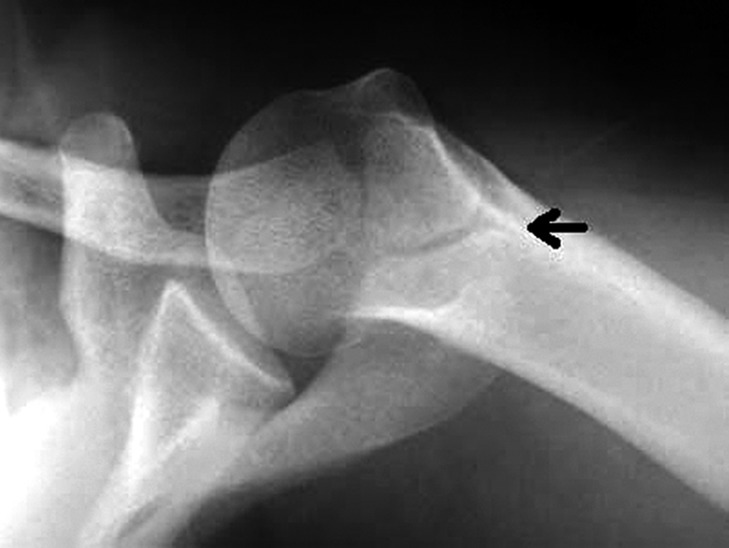
Axillary lateral radiograph demonstrating a meso‐acromion (arrow)^28^.

**Figure 4:**
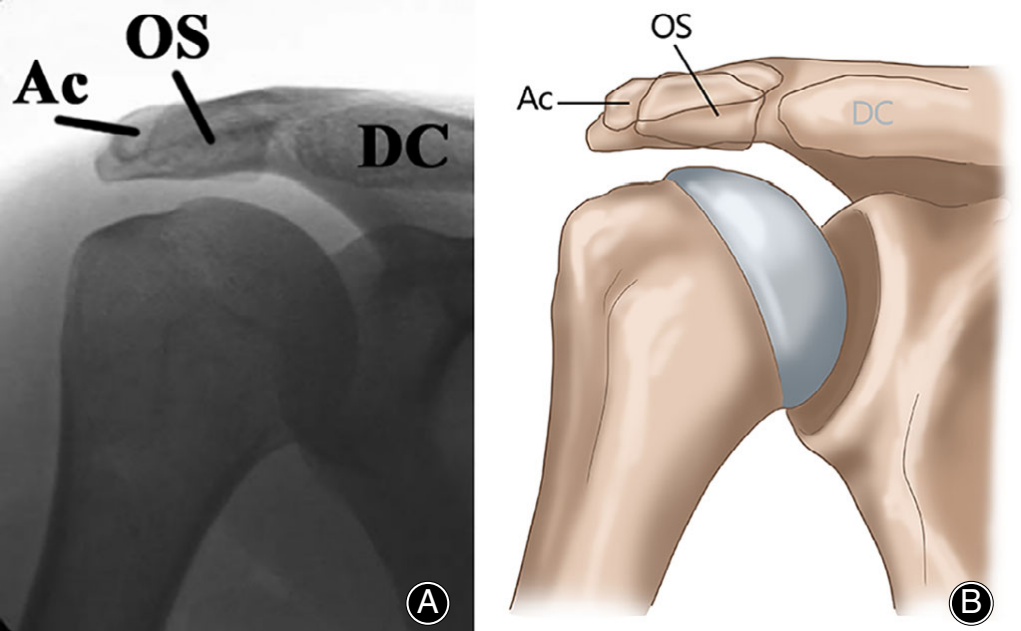
(A) Anteroposterior radiograph demonstrating the double‐density sign, with the cortical margin of a meso‐acromion (OS) superimposed over the cortical margin of the base of the acromion (Ac) at the nonunion site. The margins of this os acromiale appear smooth, sharp, and well circumscribed. DC, distal part of the clavicle. (B) Diagrammatic representation of the radiograph^28^.

Although the os acromiale usually can be seen on an axillary lateral radiograph (Fig. 3)^28^, it can be obscured by the proximal part of the humerus and be missed, in which case the double‐density sign becomes a very important and typical indication in the anteroposterior view of the shoulder (Fig. 4)^28^. In addition, ultrasound is a quick and accurate method. In Boehm *et al*.^29^, the os acromiale could be identified in all 25 patients (100%) with radiologically confirmed os acromiale. In 12 patients, an os acromiale could be identified on the contralateral side (48%). The average width of the non‐ossified space was 4.3 mm (2.5 to 7.6 mm). Three different sonographic types of bony margins of the os acromiale and the acromion exist: Type I with flat bony margins; Type II with marginal osteophytes and Type III with inverted bony margins. The authors concluded that ultrasound could give additional information about the os acromiale if radiological assessment is unclear because of superimposing bones.

**Figure 5:**
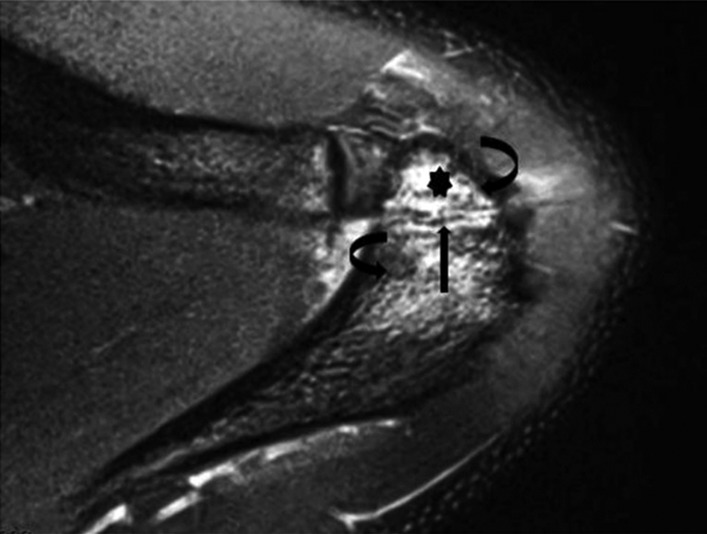
Axial proton density‐weighted fat‐saturated image of the left shoulder of a 17‐year‐old boy demonstrates an ununited ossicle adjacent to the acromion suggestive of os acromiale. There is a fluid‐like signal at the interface (arrow) and marrow edema along the opposing surfaces (curved arrows) of the distal acromial ossification center (asterisk) and the rest of the acromion^33^.

**Figure 6:**
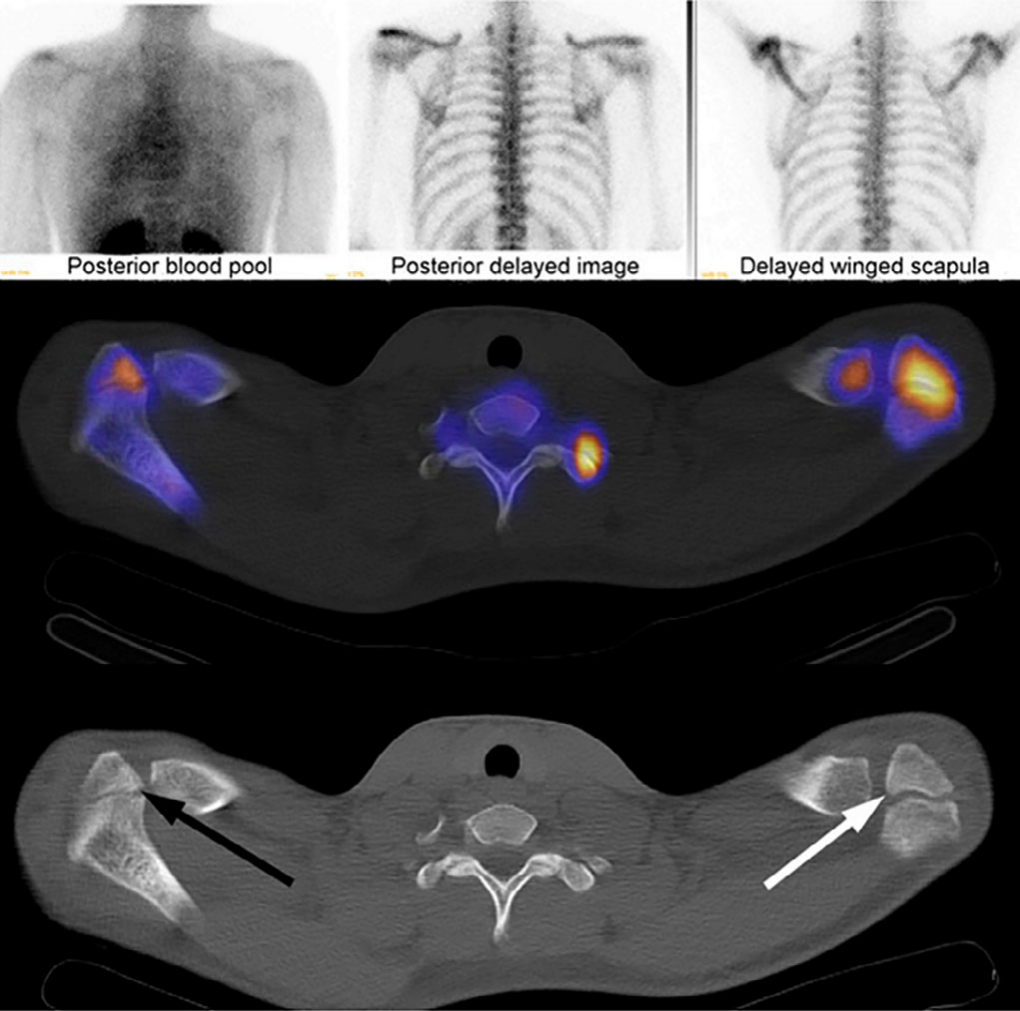
Top row shows static bone scan and blood‐pool images, middle row images shows fused SPECT/CT images, and bottom row shows attenuation correction CT. Arrows are placed at ossification centers of apophyses. Center is at meso‐acromion on the left (white arrow) but at preacromion on the right (black arrow). The right apophysis is nearly fused and has less activity than the unfused apophysis of the left acromion^34^.

The radiologic diagnosis of os acromiale remains challenging in young patients. In an adolescent patient presenting with a painful shoulder, it may be difficult to distinguish a normally developing acromion with a secondary ossification center from the early formation of an os acromiale on the basis of age alone. Therefore, MRI and SPECT–CT are receiving more and more attention^26^, ^30^, ^31^, ^32^. Winfeld *et al*.^33^ demonstrated that the unique morphologic and signal intensity characteristics of the interface between the native acromion and unfused ossification center strongly assist the ability to diagnose an os acromiale on MRI, because MRI can not only show the abnormal shape but also display the marrow edema along the opposing surfaces (Fig. 5)^33^. In the correct clinical context, a shoulder MRI of an adolescent with imaging findings consistent with os acromiale should be reported, as this may, in fact, be a sign of ossicle instability and the potential source of symptoms as well as the target of treatment strategies. Recently, Al‐faham reported on an 18‐year‐old male American football player who presented with persistent left shoulder pain without positive findings on X‐rays or MRI. SPECT/CT with 99mTc‐MDP Bone Scintigraphy was performed for further anatomic localization with the field of view restricted to the shoulders. The images demonstrated incomplete fusion of both acromion processes, which could be age‐related in this patient. However, the ossification center in the left apophysis occurred at the mesoacromion rather than at the preacromion (as on the right side) and was associated with more activity (Fig. 6)^34^, indicating ongoing osteoblastic activity likely from incomplete fusion. This finding was consistent with pain and the youth was diagnosed as having os acromiale.

### *Treatment*

Nonsurgical management: Most cases of os acromiale are asymptomatic and, thus, require no specific treatment^35^. Primary management of symptomatic os acromiale should be nonsurgical. Nonsteroidal anti‐inflammatory drugs, in conjunction with physiotherapy, are prescribed for a typical impingement treatment protocol. Subacromial and nonunion site corticosteroid injection also may be used to relieve symptoms^13^. Usually, conservative treatment should be tried for at least 6 months^15^.

Once all conservative means have failed, surgical treatment should be considered. Numerous surgical procedures have been introduced, including open or arthroscopic excision of the os fragment, ORIF with or without bone grafting, arthroscopic subacromial decompression with acromioplasty, and arthroscopically assisted reduction–internal fixation. Depending on the individual situation, various techniques have different indications. Basically, surgical techniques should focus on the os acromiale itself when the non‐healing site is unstable and painful only; however, the concomitant pathology, including rotator cuff tears or shoulder impingement, need to be solved together in some cases.

#### *Open/Arthroscopic Assisted Reduction and Internal Fixation*

**Figure 7:**
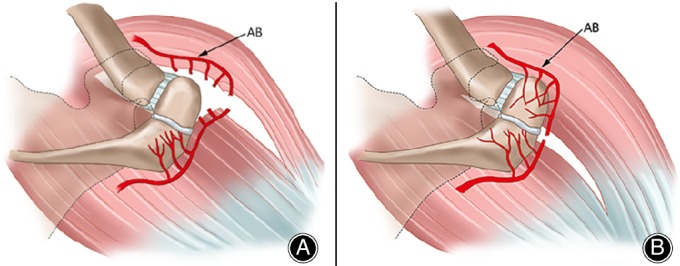
(A) Deltoid‐off approach: Terminal branches of thoracoacromial artery have been divided. Hence, unfused acromial epiphysis is devascularized. (B) Transacromial approach. Terminal branches of thoracoacromial artery remain intact. Acromial epiphysis remains vital and maintains full healing potential^36^.

As to the unstable and painful os acromiale, internal fixation can fix the fragment and relieve the pain from the pulling of deltoid; therefore, its primacy and significance are widely recognized^9^, ^21^, ^36^, ^37^, ^38^, ^39^, ^40^. Fusion is sometimes difficult to accomplish, so some surgeons^9^, ^36^, ^38^, ^40^ prefer to use a transacromial approach to preserve the terminal branches of the thoracoacromial artery (Fig. 7)^36^; furthermore, some doctors tend to use local bone graft or iliac crest bone graft to improve the fusion^39^, ^41^. During the operation, there are two key points which need to be considered. First, the sclerotic edges of the pseudarthrosis should be excised with marginal dorsal wedge cuts by use of a microsaw while preserving as much bone as possible; then the anterior portion of the acromion is fixed in a tilted upward position with a large subacromial space, which makes the acromioplasty non‐essential^21^, ^38^. Second, although the K‐wire and tension band provide stable fixation and good outcomes, nonunion and hardware discomfort are not uncommon. For these reasons, cannulated screws and the tension band technique was recommended^42^. In addition, it is reported that polyethylene sutures and stainless steel wire have similar biomechanical strength in the cannulated screw tension band fixation, which may prevent soft‐tissue problems^35^.

**Figure 8:**
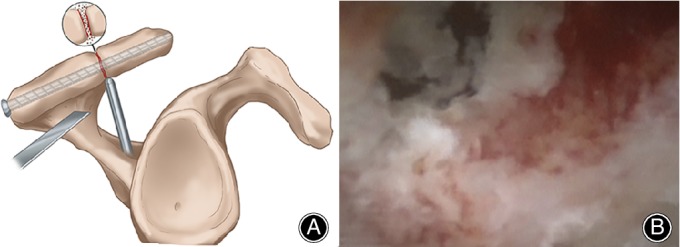
(A) Schematic drawing shows compression of the acromion and the os acromiale fragment. (B) Arthroscopic view shows compression of the acromion and the os acromiale fragment^8^.

In addition to these open surgeries, the arthroscopic technique is intended to preserve the blood supply to the os acromiale, to minimize deltoid muscle injury by avoiding its detachment, to improve the cosmetic results, and to preclude the need for hardware removal. Atoun *et al*.^8^ pointed out one arthroscopically‐assisted internal fixation with absorbable screws (Fig. 8)^8^ provided promising clinical, cosmetic, and radiologic results with high patient satisfaction. The arthroscope is introduced to the subacromial space through the same skin incision, and an evaluation of the bursal side of the rotator cuff, acromion, and os acromiale is performed. A shaver blade is introduced through a standard lateral portal (4 to 5‐cm lateral to the lateral edge of the acromion at the line of the anterior distal clavicle) and used to expose the os acromiale by removing the inferior soft tissues. After full assessment and debridement of the nonunion site, two biodegradable 4.5‐mm screws (Inion, Tampere, Finland) are used to achieve a good compression of the mesoacromion and meta‐acromion fragments. However, the biomechanical strength of biodegradable screws requires further research, and it is difficult to make a marginal dorsal wedge excision and fix the acromion at a tilted upward position, which means subacromial decompression is hard to avoid.

#### *Open/Arthroscopic Resection of Os Acromiale*

It is generally recommended that small acromial fragments should be excised, and that large ones should be fused^9^. However, the outcomes of open excision do not seem very satisfactory owing to the postoperative weakness and dysfunction of deltoid. Mudge *et al*.^43^ had eight rotator cuff tears associated with os acromiale. Six underwent fragment excision and rotator cuff repair, including suturing of the deltoid to the acromion. Four of them had excellent results postoperatively, while the remaining two had poor results. Warner *et al*.^21^ demonstrated a good outcome in one pre‐acromion excision and poor outcomes in two meso‐acromion excisions with lingering weakness and pain. Boehm *et al*.^14^ used an anterior approach releasing deltoid in all 33 patients and reconstructed the rotator cuff before treatment of the os acromiale. After repair of the cuff, the authors used one of three surgical procedures to treat the os acromiale, including excision, acromioplasty, and fusion. The constant scores were 82, 81, 81, and 84% for patients who had excision, acromioplasty, successful fusion and unsuccessful fusion, respectively. There were no statistically significant differences. The study concluded that a small mobile os acromiale can be resected, a large stable os acromiale treated by acromioplasty, and a large unstable os acromiale treated by fusion to the acromion. Even without radiological fusion, the clinical outcome can be good.

Arthroscopic excision has the possible benefit of less periosteal and deltoid attachment injury, potentially lending to better results than open excision^44^. Campbell *et al*.^45^ demonstrated no decrease in deltoid function or strength compared with the contralateral arm and found no difference in results when the excision was performed with or without a rotator cuff repair. In addition, Kawaguchi *et al*.^46^ reported a case of impingement syndrome of the left shoulder secondary to unstable meso‐acromiale, which accepted the arthroscopic excision of the unstable fragment and was successful without residual dysfunction of the deltoid muscle.

### *Disputes*

#### *Os Acromiale Combined with Shoulder Impingement or Rototar Cuff Tear*

Although the surgical management of simple os acromiale and its results have been accepted, the ideal treatment for os acromiale‐related diseases is remains controversial. In some studies, os acromiale with shoulder impingement or rototar cuff tear has been described as a depressing event. Hutchinson and Veenstra^47^ discussed the unsatisfactory results for three patients who underwent routine arthroscopic subacromial decompression. All the patients initially had a good outcome (in the first few months). However, the three patients experienced a return of their preoperative symptoms 1 year postoperatively and two of them underwent repeat surgery. Hence, the study concluded that arthroscopic subacromial decompression is probably not a solution for impingement syndrome secondary to os acromiale. Abboud *et al*.^48^ treated eight patients (os acromiale associated with rotator cuff tears) with open reduction‐internal fixation (ORIF) and open rotator cuff repair. Although all the os fragments achieved union, only three (37.5%) obtained a satisfactory result. The authors inferred that the poor results may be related to the hardware‐sourced pain even after the nonunion healed, as well as the bias in population in part (47% were involved in workers’ compensation claims). In contrast, Wright *et al*.^49^ presented an extended arthroscopic subacromial decompression. The goal of the modified arthroscopic acromioplasty was resection of adequate bone to remove the mobile anterior acromial tip. In general, this consisted of more bony resection than for the typical arthroscopic acromioplasty. All patients achieved full strength of the anterior deltoid and rotator cuff muscles by 6 months postoperatively as evaluated by manual muscle testing. At the final follow‐up (average 29 months, range from 20 to 72 months), 12 of the 13 shoulders were rated by the patients as having a satisfactory result. The study reminds us the os acromiale may not affect the strength of anterior deltoid obviously. Walch *et al*.^50^ demonstrated that preoperative acromial lesions such as os acromiale are not a contraindication to reverse shoulder arthroplasty (RSA). These patients can also acquire a good postoperative range of motion, Constant score, or subjective results compared to normal acromial patients, even without osteosynthesis of the free fragment when performing RSA. One of the possible reasons in the study was that the main part of the deltoid was still attached firmly to the spine of the scapula and clavicle and was obviously strong enough to compensate for the middle part. In the same way, Aibinder *et al*.^51^ observed that RSA did not seem to be negatively affected by the presence of an os acromiale. Inferior tilting of the unfused segment is observed in approximately one‐third of the shoulders after RSA and does not seem to change the overall outcome of the procedure. This means that even if the RSA requires particularly good function of the deltoid, healing and stability of the os acromiale are not necessary.

#### *The Relationship between Os Acromiale and Acromioclavicular Joint*

In addition to the deltoid, the articulation of os acromiale with the lateral end of the clavicle is another area of interest. Based on the review, there is only one published paper in the English literature: it investigated 211 volunteers (control group) and 33 subjects without or with os acromiale^52^. Half of the acromions of the control group (52.1%) had the articular facet of the acromioclavicular joint (ACJ) on the acromion tip, whereas in 45.4% the facet tip was located distally. In contrast, of 33 subjects with os acromiale, 18.1 and 81.1%, respectively, had the AC joint lying on or distal to the acromion tip. The author suggested that the greater the distance of the AC joint from the anterior edge of the acromion, the higher the likelihood of an os acromiale. In other words, the AC joint position was responsible for a higher predisposition to os acromiale. Because of the limited samples and lack of post and successive studies, the real cause‐and‐effect relationship between os acromiale and AC joint is still not clear. Another possible explanation is that the os acromiale affects the development of ACJ. The instability resulting from os acromiale may produce the chronic dislocation of ACJ to the posterior side, which could answer why the frequency of the mesoacromion is higher than that of the preacromion. There is no published data evaluating the association of os acromiale with degenerative change of ACJ and there is no published study assessing the stability of os acromiale following resection of lateral end clavicle for OA of ACJ. Clearly, more studies need to be done in the future.

### *Complications*

As previously mentioned, hardware‐sourced problems were often occurred after tension band fixation. Depending on the fixation technique, the incidence of nonunion after internal fixation was from 0% to 100%^37^. In addition, Boehm *et al*.^14^ showed the postoperative infection rate with two superficial infections (6%) and four deep infections (13%). One of the deep infection cases was following ORIF (5%, 1/22), one case was following open excision (17%, 1/6), and two cases were following open acromioplasty (40%, 2/5) as well. Two cases of superficial infection followed ORIF (9%, 2/22).

### *Conclusion*

Os acromiale is not a rare finding in patients with painful shoulders and the meso‐acromion type is found in clinic most frequently. There is a strong relationship between os acromiale and race, except for Korean patients, while the correlation between os acromiale and rotator cuff tears is controversial. Os acromiale in adults is easily diagnosed by X‐ray, particularly on the axillary view; however, the differential diagnosis of adolescents may require MRI or SPECT–CT. Generally, nonoperative therapy for symptomatic os acromiale should be commenced, including physiotherapy, and administration of nonsteroidal anti‐inflammatory drugs and injections. Surgical treatment is indicated after failed conservative treatment. In symptomatic patients with fixable os acromiale, the tension band technique should be used so that the anterior aspect of the acromion is elevated from the humerus head. In patients with small fragments which are unsuitable for reattachment, excision might be the best therapeutic option and can lead to good outcomes. Whether internal fixation or resection, the arthroscopic technique results in a better outcome and fewer complications, especially in older patients or athletes with overhead movement, because of the high incidence of shoulder impingement or rototar cuff tears which can be treated concurrently. In reviewing the literature, it is found that extended arthroscopic subacromial decompression, successful rotator cuff repair, and RSA could also achieve a good result in subacromial impingement, rotator cuff tear, or end‐stage cuff tear arthropathy, respectively, even without osteosynthesis of the os acromiale. In the future, a prospective study is necessary to answer this question of whether the os acromiale is related to those patients with high incidence of subacromial impingement or rotator cuff tears.
